# Rheumatic Diseases and Obesity: Adipocytokines as Potential Comorbidity Biomarkers for Cardiovascular Diseases

**DOI:** 10.1155/2013/808125

**Published:** 2013-11-26

**Authors:** Rossana Scrivo, Massimiliano Vasile, Ulf Müller-Ladner, Elena Neumann, Guido Valesini

**Affiliations:** ^1^Dipartimento di Medicina Interna e Specialità Mediche, Reumatologia, Sapienza Università di Roma, Viale del Policlinico 155, 00161 Rome, Italy; ^2^Department of Rheumatology and Immunology, University of Gießen, Kerckhoff Klinik, Benekestr 2-8, 61231 Bad Nauheim, Germany

## Abstract

Inflammation has been recognized as a common trait in the pathogenesis of multifactorial diseases including obesity, where a low-grade inflammation has been established and may be responsible for the cardiovascular risk related to the disease. Obesity has also been associated with the increased incidence and a worse outcome of rheumatoid arthritis (RA) and osteoarthritis (OA). RA is characterized by systemic inflammation, which is thought to play a key role in accelerated atherosclerosis and in the increased incidence of cardiovascular disease, an important comorbidity in patients with RA. The inflammatory process underlying the cardiovascular risk both in obesity and RA may be mediated by adipocytokines, a heterogeneous group of soluble proteins mainly secreted by the adipocytes. Many adipocytokines are mainly produced by white adipose tissue. Adipocytokines may also be involved in the pathogenesis of OA since a positive association with obesity has been found for weight-bearing and nonweight-bearing joints, suggesting that, in addition to local overload, systemic factors may contribute to joint damage. In this review we summarize the current knowledge on experimental models and clinical studies in which adipocytokines were examined in obesity, RA, and OA and discuss the potential of adipocytokines as comorbidity biomarkers for cardiovascular risk.

## 1. Introduction 

Adipocytokines are a very heterogeneous group of soluble proteins showing pro- or anti-inflammatory effects. Many adipocytokines are mainly secreted by the adipocytes of white adipose tissue (WAT), which is nowadays considered a major endocrine organ through the capability of secreting adipocytokines [1]. The most widely studied adipocytokines are leptin, adiponectin, resistin, and visfatin. Leptin plays a key role in the regulation of appetite and body weight and in the modulation of immune responses. Circulating leptin concentrations are increased in obesity, and these increased levels are associated with the development of inflammation, insulin resistance, and subclinical coronary atherosclerosis [2, 3]. Elevations in resistin and visfatin are also associated with increased inflammation, insulin resistance, and cardiovascular risk [2, 4]. In contrast, adiponectin is an anti-inflammatory adipocytokine, and increased concentrations are inversely associated with obesity, insulin resistance, and cardiovascular risk [2]. Hence, all of these adipocytokines are actively involved in obesity, but the precise mechanism needs to be defined. Interestingly, WAT hosts a special microenvironment during obesity, enriched with many immune cell populations interacting with adipocytes [1], and this strict interaction may sustain the pathways linking metabolism and the immune system. Indeed, when adipose tissue inflammation and dysfunction have developed, adipokine secretion is significantly changed towards a proinflammatory, diabetogenic, and atherogenic pattern [5, 6]. Recently, the identification of biomarkers gained increased attention in many fields of medicine, including rheumatology. Per the definition of the working group of the National Institutes of Health (NIH), a biomarker is assumed to be “a characteristic that is objectively measured and evaluated as an indicator of normal biological processes, pathogenic processes, or pharmacologic responses to a therapeutic intervention” [7]. These features may help physicians to recognize disease susceptibility, prognosis, and therapeutic response that are vital issues when assessing chronic diseases, including rheumatic conditions. However, some biomarkers may be disease related, such as anti-citrullinated protein/peptide antibodies (ACPA) in rheumatoid arthritis (RA), while others appear to be inflammation dependent, and in this perspective we may consider several adipocytokines. Since obesity may be associated with other chronic conditions, including RA, whose onset and outcome are affected by obesity [8, 9], and osteoarthritis (OA) [10], the purpose of this review is to summarize the literature related to adipocytokines in obesity, RA, and OA and to discuss whether they may be considered as comorbidity biomarkers for cardiovascular risk, potentially worsening the outcome of these diseases. The literature search relied on PubMed (from January 1, 1990, through March 31, 2013) and was limited to original research involving animal models and human subjects published in English and having abstracts. The articles were identified using headings consisting of a combination of at least two among “rheumatoid arthritis, osteoarthritis, obesity, cardiovascular risk, adipocytokine, biomarker, adiponectin, leptin, resistin, and visfatin.”

## 2. Obesity

Overweight and obesity are defined by the World Health Organization (WHO) as abnormal or excessive fat that accumulates and presents a risk to health [11]. Over the past years, obesity has become epidemic in many countries and has been recognized as a challenge for public health since it may contribute, together with abdominal fat distribution, to the individual risk for type 2 diabetes, dyslipidemia, fatty liver disease, chronic subclinical inflammation, hypertension, and cardiovascular disease [12–14]. In the past decades, advances in obesity research have led to the recognition that adipose tissue is an active endocrine organ that secretes several bioactive proteins termed adipocytokines [1]. In an autocrine and paracrine manner, adipocytokines contribute to the modulation of adipogenesis, immune cell migration into adipose tissue, and adipocyte metabolism and function [5, 6]. Hence, they may be involved in the pathogenesis of obesity and the role of some of them (leptin, adiponectin, resistin, and visfatin) has been extensively studied in the disease. The main findings related to these studies are summarized in Table 1.

### 2.1. Experimental Models

Leptin mRNA levels were increased in mice adipose tissue after the exposure to proinflammatory cytokines [15]. In addition, leptin has been shown to acutely decrease in mice with caloric restriction and increase with refeeding and also induces anorexigenic factors [16]. Also adiponectin seems to regulate metabolic pathways in animal models. In fact, treatment with adiponectin decreases hyperglycaemia and plasma levels of free fatty acids and improves insulin sensitivity in obese animals [17], while adiponectin-deficient mice develop diet-induced insulin resistance on a high-fat, high-sucrose diet [18].

Differently from leptin and adiponectin which are mainly produced by adipocytes, visfatin and resistin are primarily secreted by the cells of immune system [19, 20]. Visfatin is a product of the pre-B cell colony enhancing factor (PBEF) gene, subsequently identified as the extracellular form of the enzyme nicotinamide phosphoribosyltransferase (NAMPT), believed to mimic insulin function [21]. Plasma visfatin concentration was increased during the development of obesity in an experimental model of obesity-associated insulin resistance [22]. Resistin is a 12 kDa polypeptide that was initially implicated in the pathogenesis of obesity-associated insulin resistance and type 2 diabetes mellitus in mouse models [23]. Mice with chronic hyperresistinemia exhibit modest fasting hyperglycemia and glucose intolerance, associated with increased hepatic glucose production in the setting of hyperinsulinemia, suggesting that chronic hyperresistinemia leads to impairment of glucose homeostasis [24].

### 2.2. Clinical Studies

In humans, leptin primarily acts on hypothalamic neurons resulting in anorexia and weight reduction [25]; in particular, serum levels decrease with fasting [26, 27] and increase during hyperinsulinemia [28]. Furthermore, there may be a direct link between circulating leptin concentrations and increased cardiovascular risk since this adipocytokine may enhance platelet aggregation and arterial thrombosis, promote angiogenesis, impair arterial distensibility, and induce proliferation and migration of vascular smooth muscle cells [29]. In contrast, circulating adiponectin levels tend to be low in morbidly obese patients and increase with weight loss and with the use of thiazolidinediones, which enhance the sensitivity to insulin [30, 31]. However, different isoforms of adiponectin have been recognized [32], which may have different effects: low, middle, and high molecular weight isoforms (LMW, MMW, and HMW) and globular adiponectin. A protective role of HMW adiponectin against the development of obesity, insulin resistance, glucose intolerance, diabetes mellitus, hypertension, metabolic syndrome, atherosclerosis, and cardiovascular disease [33–37] and a negative role of LMW adiponectin on diabetes mellitus and cardiovascular disease have been described [38–41]. In humans, circulating visfatin levels are increased in diabetic subjects and are closely correlated with WAT accumulation [22, 42]. However, the current data on visfatin levels in humans are controversial in many aspects: the effective cellular source of visfatin in visceral fat in obese individuals, the possible influence of gender in its production, the association between visfatin mRNA expression in visceral fat mass with the body mass index (BMI), and the correlation between plasma levels with the total amount of visceral fat and plasma lipids [21]. A possible explanation for these conflicting findings may reside in the fact that, although visceral fat may be a central source of visfatin, the producing cells may be mainly other cells of the adipose tissue, not adipocytes. There could also be a stronger influence of inflammatory cells/stage on systemic visfatin level than on other adipocytokines, but also polymorphisms in coding regions of the genes may be responsible for different effects in the different population considered. Regarding the gender differences, hormones or even the different dispositions of adiposity in male and female may play a role, while the influence on lipid profile is probably linked to intracellular enzymatic function in nicotinamide adenine dinucleotide (NAD) synthesis [43]. Similarly, studies in humans have shown conflicting results when examining resistin levels in obese and lean subjects, the adipose resistin expression, or the role of resistin in the development of insulin resistance [44–51]. In one of these, serum resistin levels were higher in female patients than in males [45]. Hence, as for visfatin, additional research is necessary to better define its role in the pathogenesis of obesity. Considering that visfatin and resistin share a common origin, mainly linked to the cells of the immune system and not adipocytes, it is likely that their prevalent activity is devoted to immunomodulation rather than control of metabolism and lipid profile. This may sustain the mixed results so far obtained.

Overall, it has been demonstrated that adiposity is associated with increased production of proinflammatory molecules, whereas reduced adiposity is associated with decreased concentration of proinflammatory and increased concentration of anti-inflammatory molecules [52]. All these findings suggest that altered adipocytokine secretion may represent a link between adipose tissue dysfunction in obesity and metabolic and cardiovascular obesity-related disorders. Leptin, adiponectin, visfatin, and resistin are important modulators of glucose metabolism because they may primarily contribute to altered appetite and satiety, impaired insulin sensitivity or secretion, and to inflammation. Hotamisligil et al. first discovered the existence of an inflammatory state involving adipose tissue and its potential role in obesity by demonstrating the secretion of TNF by the adipose tissue [53]. In addition to adipocytes, macrophages in human adipose tissue may contribute to enhancing the obesity-related “low-grade” chronic inflammation [14]. The action of the inflammatory molecules may represent the molecular link between adipose tissue and the cardiovascular complications of obesity [14]. Despite these considerations, we believe that adipocytokines cannot still be included as biomarkers of cardiovascular risk in obese subjects: although they may have a clinical relevance as biomarkers for fat mass, more focused studies are needed to evaluate their potential in the assessment of cardiovascular function in obese individuals.

## 3. Rheumatoid Arthritis 

RA is an autoimmune disease affecting 0.5–1% of the adult population with potential destructive effects on diarthrodial joints and often burdened by comorbidities, particularly in the cardiovascular system. Indeed, people with RA die prematurely, mostly due to higher rates of cardiovascular events [54]. Concentrations of adipocytokines have generally been reported to be higher in patients with RA than in control subjects [55–57], and it is supposable that they may also play a role in the increased cardiovascular risk since obesity is also associated with this unfavorable outcome [58]. Here we summarize the available experimental and clinical data in which adipocytokines were examined, discussing their potential role as biomarkers of cardiovascular risk. In addition, an overview of the central findings of these studies is reported in Table 2.

### 3.1. Experimental Models

Adiponectin is one of the most studied adipocytokines in the context of this review. It has been shown to be secreted not only by WAT, but also locally by osteoblasts and hepatocytes during inflammatory processes [59–61] and by RA synovial fibroblasts (RASF) [62]. In contrast to findings in other inflammatory diseases [18, 63], both RA synovial tissue and articular adipose tissue were a significant source of adiponectin, capable of stimulating RASF to produce IL-6 and prometalloproteinase-1, a finding that supports an active role of this adipocytokine in the pathogenesis of RA [62]. These results were confirmed in recent studies, where adiponectin stimulation induced the secretion of chemokines and proinflammatory cytokines by fibroblasts and other immune cells and of matrix metalloproteinases (MMP) by fibroblasts and chondrocytes in synovial tissue from RA patients [32, 64]. Furthermore, several findings *in vitro* suggest that adiponectin may actively promote RA progression as it induces the secretion of proinflammatory molecules (e.g., IL-6, COX-2), chemokines (e.g., IL-8, MCP-1), and matrix-degrading enzymes (e.g., MMP-3) *in vitro* [64, 65]. Hence, adiponectin seems to have a strong proinflammatory effect in RA, which may also sustain the increased cardiovascular risk observed in some patients. In particular, increased levels of proinflammatory cytokines, including IL-6, may directly contribute to the mechanisms of change in the insulin sensitivity in different adipose depots [14]. Interestingly, insulin resistance is increased in patients with RA and is associated with accelerated coronary atherosclerosis [66].

The other well-known adipocytokines, leptin, resistin, and visfatin show also predominantly proinflammatory properties similar to the local effects described for adiponectin. In antigen-induced arthritis models, leptin-deficient mice developed less severe arthritis with lower mRNA levels of proinflammatory cytokines compared with control mice and had reduced inflammation [67]. Furthermore, mice with a mutation in the gene encoding leptin or the gene encoding the leptin receptor both displayed obese phenotypes and various defects in cell-mediated and humoral immunity [68], thus providing a molecular mechanism sustained by leptin linking metabolic processes and immune dysfunctions.

Resistin showed a strong upregulation of TNF and IL-6 expression by human peripheral blood mononuclear cells. It induced arthritis onset after injection into the joints of healthy mice, and the frequency of arthritis increased in a dose-dependent manner [69]. Visfatin was shown to be involved in RASF activation by triggering fibroblast motility and promoting high amounts of chemokines, proinflammatory cytokines, and MMPs synthesis by these cells [70]. These results show a strong contribution of visfatin to synovial inflammation in RA, suggesting that this may be a potential biomarker for RA.

### 3.2. Clinical Studies

In patients with RA a low BMI appears to be associated with a significant risk of cardiovascular death, even after adjustment for cardiac history, smoking, diabetes mellitus, hypertension and malignancy [71]. This may be due to the state of rheumatoid cachexia, typical for RA patients, which show characteristic low muscle and high fat mass. In addition, considering that increased central adiposity is common in RA [72] and is associated with insulin resistance [73], the role of adipocytokines in RA inflammation appears captivating. In fact, most evidence suggests that classic risk factors do not explain excess vascular disease in RA, and systemic inflammation independently predicts cardiovascular events in men and women with or without existing heart disease [74].

The diverse isoforms of adiponectin have different potencies to modulate gene expression of RASF [32] or in part even contrary effects [75, 76].

Available data on RA suggest that adiponectin is associated with disease progression [77–79], probably because adiponectin may stimulate osteoclast differentiation via increasing RANKL and decreasing osteoprotegerin [80] and may up-regulate vascular endothelial growth factor and MMPs [65]. Another possible underlying mechanism could be the effect of IL-6 on ACPA-producing B cells, because IL-6 is a well-known growth factor for B cells and has been shown to play a role in mouse models of antibody-mediated arthritis [81, 82]. However, adiponectin and leptin serum levels from RA patients were neither associated with clinical and serological features of inflammation nor were they down-regulated after 12 weeks of anti-TNF treatment [83], in contrast with findings shown *in vitro* [62, 64, 65]. Furthermore, early and chronic RA patients had higher plasma adiponectin levels compared to healthy controls, but they were lower than those of patients with OA [84]. A possible explanation for the discrepancy of experimental and clinical findings could be due to relevance of metabolic and systemic regulation of adiponectin over the local phenomenon. It may also be a consequence of the individual adiponectin isoforms with different potencies to modulate gene expression of RASF locally as well as systemically, suggesting that some of them are more detrimental in RA than others, even if no opposing effects in the setting of RA pathophysiology were found [32]. While adiponectin levels were associated with radiographic damage and RA progression [78, 84], the levels decreased as visceral fat area increased. Hence, this adipocytokine might be a mediator of the inverse association of visceral fat with radiographic damage [85]. Consistent with these results showing an inverse relation between severity of RA and adipose tissue, a high BMI was inversely associated with the amount of joint destruction in patients with early RA, although only in those with a positive ACPA status [86]. A peculiar feature of adiponectin physiology is that circulating levels diminish as adiposity increases, with the highest levels in subjects with the lowest fat mass [87]. Hence, considering the detrimental effects on the joint, adiponectin becomes an excellent candidate to mediate the inverse relationship between increasing adiposity and radiographic damage.

Data on leptin in RA are likewise controversial regarding serum levels: in some studies RA patients and controls with a similar body fat content and BMI or when adjusted for BMI did not differ with respect to systemic leptin concentrations [88–91], while in others they were higher than controls [55, 92–96]. Furthermore, these ambiguous results were not limited to serum/plasma concentrations since higher leptin levels correlated with disease activity or clinical features [55, 56, 95, 96], whereas other studies did not confirm these findings [90, 91, 94]. Moreover, in one report an inverse correlation with C-reactive protein (CRP) and IL-6 levels was described [89]. However, leptin may have a protective effect against joint damage in RA, as it was hypothesized in the study by Rho et al. [95]. Here, leptin concentrations were found to be associated with reduced radiographic joint damage, particularly after adjustment for measures of inflammation (disease activity score 28, IL-6, CRP). Recently, a similar conclusion was made from the observation that synovial fluid (SF) leptin levels were lower in nonerosive patients, suggesting that a local leptin consumption may be protective against erosions [96], as previously described [69]. In favour of this suggestion is the knowledge that leptin induces IL-1 receptor antagonist production [97], and treatment of RA patients with IL-1 receptor antagonist has been proved to stop the joint destructive process [98]. Higher leptin levels were also associated with insulin resistance in RA, although they paradoxically attenuated the effect of insulin resistance on severity of coronary calcification [99]. This finding was interpreted by the authors as an overall effect of leptin on atherosclerosis mediated through interactions with other risk factors for atherosclerosis, rather than an independent effect in RA. Alternatively, high leptin concentrations could reflect a feedback mechanism to improve insulin resistance and also ameliorate its effects on atherosclerosis in RA [99].

Resistin levels were found to be increased in the serum and accumulated in the inflamed joints of RA patients [57, 69, 100]. Furthermore, they were found to be predictive with regard to radiological damage, irrespective of CRP levels or ACPA status, in a cohort of patients treated with adalimumab. In this study resistin levels declined after long-term adalimumab or glucocorticoid treatment in parallel with a decrease of inflammatory markers and also the lipid profile was ameliorated [101]. Hence, resistin seems to have a definite pathophysiological role in RA inflammation and damage, while the potential involvement in cardiovascular risk in these patients has not been investigated.

**Figure 1:**
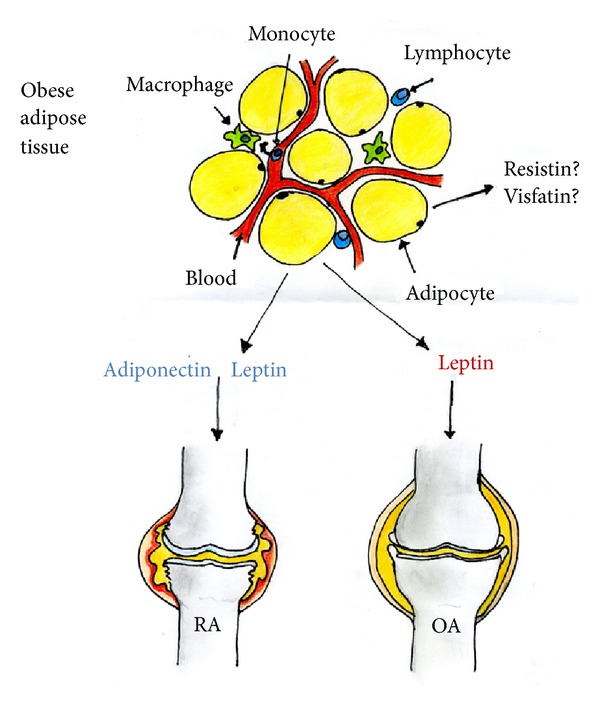
The white adipose tissue (WAT) is considered a major endocrine organ through the capability of secreting adipocytokines. In obese individuals, WAT hosts many immune cell populations interacting with adipocytes. Obesity is a risk factor for both rheumatoid arthritis (RA) and osteoarthritis (OA), and it is likely that some adipocytokines are involved in the pathogenesis of these two diseases. In RA, serum adiponectin levels were associated with radiographic damage and decreased as visceral fat area increased; leptin levels were associated with insulin resistance. In OA, leptin levels were associated with increased levels of bone formation biomarkers and erosive disease, and a positive correlation with the body mass index was also observed. These adipocytokines may be involved in the increased cardiovascular risk observed in RA and OA patients. Conversely, the role of resistin and visfatin is still controversial.

In conclusion, both experimental and clinical data show a strong proinflammatory potential for these adipocytokines in RA, although many data remain controversial. Adiponectin and leptin are the two adipocytokines showing a potential for being comorbidity biomarkers of cardiovascular risk in RA patients (see Figure 1). Adiponectin levels were associated with radiographic damage and RA progression. However, adiponectin seems to be a mediator of the inverse association of visceral fat with radiographic damage that may be related to the state of rheumatoid cachexia, characterized by low muscle mass and high fat mass. Indeed, it was observed that serum adiponectin concentration decreased as visceral fat area increased, leading to the inhibition of radiographic damage progression [78, 85]. Also leptin may be involved in cardiovascular risk, due to the association of serum levels with insulin resistance and the effect on atherosclerosis [99].

## 4. Osteoarthritis

OA in general develops progressively over several years, although symptoms might remain stable for long periods, and indeed it becomes more common with age. The diagnosis relies on clinical and radiological features since nearly half of the patients with radiological features have no symptoms and vice versa [102]. The disease is characterized by biomechanical and biochemical changes in the cartilage, subchondral bone, and synovial tissue [102]. Obesity is doubtless a very relevant etiologic factor for OA due to the overload effect on joint cartilage. In fact, chondrocytes and osteoblasts are sensitive to pressure through the presence of mechanoreceptors [103], whose activation may trigger both the inhibition of matrix synthesis and cartilage degradation. However, a positive association between OA and obesity has also been found for non-weight-bearing joints [104] suggesting that, in addition to local overload, systemic factors contribute to joint damage. The central role of inflammatory processes in OA supports this view, and it is relevant to note that the risk of hand OA is about 2-fold in obese people as compared with normal-weight subjects [105]. The inflammatory mediators responsible for this observation in OA probably also include adipocytokines. Therefore, they were recently investigated for their utility in providing diagnostic or prognostic clues as biomarkers for OA. An overview of the key findings of studies investigating adipocytokines in OA is reported in Table 3.

### 4.1. Experimental Models

Leptin is the most studied adipocytokine in OA experimental models, with recent studies supporting its pathogenetic role. It was demonstrated that leptin has a catabolic role on cartilage metabolism, inducing collagen release from bovine cartilage and stimulating MMP expression in chondrocytes cultured with WAT-conditioned media taken from fat pads from OA patients [106]. Other findings support a role of leptin in cytoskeletal remodeling, which is also implicated in OA pathogenesis, since leptin-treated human chondrocytes showed an activated Rho/ROCK pathway signaling leading to change of cell shape and stress fiber formation [107]. However, these data are not consistent with a previous study in which physiologic doses of leptin were not able to affect matrix biosynthesis, proteoglycan breakdown, or nitric oxide production *in vitro* in cartilage explants from mice with OA [108]. Hence, leptin may be a secondary mediator of cartilage degeneration in OA. Indeed, the proinflammatory effects of leptin are apparent at superphysiologic concentrations: OA increases the expression of leptin and leptin receptors in chondrocytes from OA samples [109], suggesting that physiologic levels of leptin may mediate the production of inflammatory mediators in osteoarthritic but not normal tissue.

As in RA, adiponectin seems to drive proinflammatory effects in RASF and adipose tissue adipocytes [62]. Recently, an increased secretion of MMP-3 in cultured human chondrocytes through its receptor AdipoR1 was found, contributing to cartilage destruction [110]. Furthermore, the pro-destructive effect of adiponectin in OA was shown in another study, in which both AdipoR1 and AdipoR2 were significantly higher in lesional than in nonlesional areas of cartilage obtained from OA patients at the time of knee-replacement surgery [111]. In addition, adiponectin was shown to induce nitric oxide synthase, IL-6, MMP-3, MMP-9, and MCP-1 in murine ATDC5 chondrogenic cell lines [112]. Only one report demonstrated a protective effect of adiponectin through the upregulation of tissue inhibitor of metalloproteinase (TIMP)-2 and downregulation of IL-1*β*-induced MMP-13 in chondrocytes [113]. In the same study, the percentage of HMW per total adiponectin in SF was lower than that in plasma, while that of the examer form (MMW) in SF and plasma, and the trimer form (LMW) was higher in SF [113]. Indeed, adiponectin stimulation increased protein secretion in OA fibroblasts to a much lesser extent than in RA [32, 64] suggesting that, as observed in RA, some adiponectin isoforms may be more detrimental than others, but also that OA fibroblasts show in general a weaker response to adiponectin stimuli than RASF. Also visfatin was shown to be involved in OA catabolism: chondrocytes produce visfatin, and stimulation of normal chondrocytes with visfatin decreases the synthesis of prostaglandins [114]. However, in human OA chondrocytes, visfatin inhibits the function of IGF-1, a well-known growth factor for several matrix components, producing a resistance to IGF-1 which negatively regulates matrix synthesis [115]. Limited data are available for resistin. The levels of this adipocytokine were measured in paired SF and serum samples from patients following joint injury and its expression was studied and found by immunohistochemistry in synovial tissue from healthy and OA donors [116]. Considering these data, we can conclude that especially adiponectin, leptin, and visfatin can promote cartilage catabolism and may have a role in the pathophysiology of OA. Current evidence is too scant for resistin to draw definite conclusions.

### 4.2. Clinical Studies

There is some evidence that the infrapatellar fat pad, also known as Hoffa's fat pad, is an important source of several central adipocytokines such as leptin, adiponectin, and resistin in OA patients [117]. In particular, the stimulation of human infrapatellar fat pad obtained from OA patients with IL-1*β* induced a 10-fold increase in leptin mRNA expression [118]. Furthermore, in patients with knee OA studied for 2 years, baseline serum levels of leptin were associated with increased levels of bone formation biomarkers [119]. The soluble receptor of leptin was associated with reduced levels of bone formation biomarkers and increased cartilage volume loss assessed by magnetic resonance imaging. In this study, adiponectin and resistin were not significantly associated with bone formation biomarkers [119]. In addition, leptin seems to be locally involved in joint erosion in OA since SF concentrations were significantly higher in OA patients compared to controls. Importantly, leptin levels were highest in patients with more severe disease [120], suggesting that SF levels could be used as an effective biomarker for quantitative detection of OA. Recent findings showed the association of higher serum leptin levels with increased odds of both prevalent and incident knee OA in a cohort of mid-life women [121]. Furthermore, a positive correlation between the BMI of OA patients and serum levels of leptin was found, whereas no correlation was detectable with age, disease duration, and visual analogue pain scale for the lower-limb afflicted patients and stage of disease [91]. All these findings strongly support a major role of leptin in the pathogenesis of OA and the potential utility as a biomarker for OA risk.

As observed in experimental models, also in humans the role of adiponectin appears controversial in OA. In patients with knee OA, plasma concentrations of adiponectin were significantly higher than those in SF and both plasma and SF levels inversely correlated with disease severity [122]. These results, showing a protective effect, were indirectly confirmed by the observation that patients with the highest levels of adiponectin had a decreased risk of hand OA progression while no association for leptin and resistin was found [123]. On the other hand, plasma adiponectin levels were found to be significantly higher in OA patients than in healthy controls in another study [84], and higher levels were also observed in female patients with erosive hand OA in comparison to those with nonerosive disease [124]. Finally, plasma adiponectin levels were higher in patients undergoing total knee replacement surgery than in patients with less severe disease [125].

Along this line, higher serum levels of resistin but not adiponectin were found in patients with radiographic subchondral erosion than in nonradiographic hand OA patients [126], while in another study involving 172 subjects no association between resistin and adiponectin serum levels with cartilage damage was found [127]. Therefore, data on adiponectin and resistin are conflicting, making the possibility to consider them as valuable biomarkers of OA hard, while data on leptin in this regard are consistent. Since leptin is almost exclusively secreted from adipocytes and obesity is associated with increased leptin serum concentrations which potentially contribute to insulin resistance and metabolic syndrome [29], this adipocytokine deserves further attention as potential comorbidity biomarker of cardiovascular risk (see Figure 1).

## 5. Conclusions

RA and OA are two epidemiologically relevant diseases leading to articular damage and disability, whose outcome can be heavily affected by comorbidities, particularly in the cardiovascular system. On the other hand, obesity is suggested to be the underlying cause of the metabolic syndrome which results in a 2- to 3-fold increase in cardiovascular risk. Obesity is also a risk factor for RA and OA, and this observation has captured attention on WAT as an immunomodulatory endocrine organ, due to the capability of secreting adipocytokines. Some of these are probably involved in the pathogenesis of RA and a possible role in the increased cardiovascular risk observed in these patients cannot be excluded, considering that increased central adiposity is common in RA. Likewise, OA does not cause death directly, but, limiting mobility and physical activity, it increases the risk of obesity and cardiovascular disease. Again, the role of adipocytokines, acting independently of mechanical stress, may be relevant and influence the prognosis.

Despite these considerations, studies evaluating adipocytokines in RA and OA have shown controversial results both in experimental models and human diseases with regard to serum/plasma levels and association with severity of disease. Focusing on the context of this review, related to the implication of adipocytokines for the cardiovascular risk, the only speculations may be done on adiponectin and leptin (see Figure 1): in RA, the first was shown to be reduced in obese patients or in those with rheumatoid cachexia and inversely correlated with radiographic damage [78, 84]; the latter was associated with insulin resistance [99], but the consequences of these association have not been further studied. Leptin seems to be a promising biomarker also for OA patients, due to its involvement in disease pathogenesis and obesity.

Hence, we believe that adipocytokines cannot be currently included in the clinical practice evaluation of RA and OA patients. Although their potential use as comorbidity biomarkers of cardiovascular risk may be of interest, a specific investigation is required due to the limitations of the data currently available.

## Figures and Tables

**Table 1:** Overview of main experimental and clinical data on adipocytokines in obesity.

Adipocytokine	Model	Finding(s)	Authors
Adiponectin	Rhesus monkeys (plasma, adipose tissue)	Levels reduced in obese and diabetic animals Levels decreased in parallel to the progression of insulin resistance No association between plasma levels and mRNA levels in adipose tissue	Hotta et al. [17]
	Knockout mice	Delayed clearance of free fatty acid in plasma, high plasma TNF levels and mRNA levels in adipose tissue, severe diet-induced insulin resistance, and low levels of fatty-acid transport protein 1 mRNA in muscle	Maeda et al. [18]
	Murine (plasma, adipose tissue) Human (plasma)	Plasma concentrations significantly increased by administration of TZDs in insulin resistant animals; adiponectin mRNA expression normalized/increased by TZDs in adipose tissues Plasma concentrations significantly increased by administration of TZDs in insulin resistant subjects	Maeda et al. [30]

Leptin	Murine (serum, adipose tissue)	Levels increased after exposure to proinflammatory cytokines Leptin mRNA expression in fat increased after exposure to proinflammatory cytokines	Sarraf et al. [15]
	Murine (serum)	Acute decrease after caloric restriction and increase after refeeding	Ahima et al. [16]
	Human (serum)	Levels higher in obese than in normal-weight volunteers and reduced after fasting; positive correlation with serum insulin and plasma glucose	Boden et al. [26]
	Human (serum)	Levels increased dose-dependently by hyperinsulinemia	Boden et al. [28]
	Human (serum)	Overfeeding and weight gain associated with elevation of leptin levels	Kolaczynski et al. [27]

Resistin	Murine (serum)	Fasted blood glucose higher in resistin-transgenic mice than in their nontransgenic littermates, glucose tolerance impaired in hyperresistinemic mice	Rangwala et al. [24]
	Human (serum, adipose tissue)	Levels elevated in obese than lean subjects, positive correlation with BMI	Degawa-Yamauchi et al. [45]
	Human (serum)	Levels not correlated with markers of adiposity, in females higher levels than males, no significant difference in levels after fasting and/or leptin administration	Lee et al. [46]
	Human (serum, adipose tissue)	Levels not different among non-obese and obese diabetic subjects, strong correlation between serum levels and resistin mRNA expression from abdominal adipose tissue	Heilbronn et al. [47]
	Human (serum)	Levels positively associated with percent body fat, not associated with fasting glucose, insulin levels, whole-body insulin sensitivity, basal hepatic glucose output, hepatic glucose output during low-dosage insulin infusion of a hyperinsulinemic clamp, or acute insulin secretory response	Vozarova de Courten [48]

Visfatin	Murine (mesenteric fat, plasma) Human (plasma)	Levels increased during development of obesity Levels elevated in diabetes	Chen et al. [22]
	Murine Human	Recombinant visfatin induced circulating IL-6 Recombinant visfatin induced the production of cytokines and modulated cytokine gene expression in PBMCs and induced chemotaxis in monocytes and B cells	Moschen et al. [42]

TZDs: thiazolidinediones; TNF: tumor necrosis factor; PBMCs: peripheral blood mononuclear cells; BMI: body mass index.

**Table 2:** Overview of main experimental and clinical data on adipocytokines in RA.

Adipocytokine	Model	Finding(s)	Authors
Adiponectin	Human (synovial tissue)	Strongly expressed in synovium and adipose tissue Stimulates the release of IL-6 and pro-MMP-1 by RASF	Ehling et al. [62]
	Human (serum)	Levels significantly higher in patients with severe disease evaluated by radiographic changes	Ebina et al. [77]
	Human (SF, synovial tissue)	Levels in SF significantly higher than in OA patients Stimulates the production of proinflammatory mediators by RASF at the same level as IL-1*β*	Choi et al. [65]
	Human (serum)	Association of serum levels with radiographic progression (stronger in patients with longer disease duration) Levels decreased as visceral fat area increased	Giles et al. [85]
	Human (plasma)	Higher levels compared to healthy controls No difference in patients with early and chronic disease	Laurberg et al. [84]
	Human (synovial tissue)	Induces gene expression and synthesis of proinflammatory mediators in RASF, lymphocytes, endothelial cells, chondrocytes	Frommer et al. [64]
	Human (serum)	Association of average levels with radiographic progression, especially in certain subgroups (women, BMI < 30 kg/m^2^, baseline biological drugs)	Giles et al. [78]
	Human (serum)	Association with proinflammatory cytokines Levels positively associated with radiographic progression over 4 years Negative correlation with BMI	Klein-Wieringa et al. [79]
	Human (synovial tissue)	Secretion of proinflammatory mediators mostly induced by HMW/MMW-enriched adiponectin, weakest response seen with trimeric form	Frommer et al. [32]

Leptin	Human (serum)	Levels not different from those in healthy controls Positive correlation with percent body fat in patients and controls	Anders et al. [88]
	Human (serum)	Levels higher than controls	Salazar-Páramo et al. [92]
	Murine (synovial tissue, serum)	Less severe arthritis and lower mRNA levels of proinflammatory cytokines in leptin-deficient mice	Busso et al. [67]
	Human (plasma, SF)	Plasma levels higher than controls Plasma levels higher than matched SF MTX treatment associated with high plasma levels	Bokarewa et al. [93]
	Human (plasma)	Levels not different from those in healthy subjects Levels inversely correlated with CRP and IL-6 levels at baseline and not modified after a short-course of anti-TNF treatment	Popa et al. [89]
	Human (serum)	Tendency to higher levels than controls Strong correlation with fat mass	Toussirot et al. [94]
	Human (plasma)	Levels higher than controls Positive correlation with CRP	Otero et al. [55]
	Human (plasma, SF)	Plasma levels not different from those in healthy controls Positive correlation of plasma levels with BMI in patients and controls No correlation of plasma and SF levels with disease activity	Hizmetli et al. [90]
	Human (serum)	Levels not different from those in OA patients Positive correlation between serum levels and BMI No correlation between serum levels and disease activity	Wisłowska et al. [91]
	Human (serum)	Higher mean levels in patients with high activity Levels significantly correlated with disease activity	Lee et al. [56]
	Human (serum)	Levels higher than controls Positive correlation between serum levels and BMI	Rho et al. [95]
	Human (serum)	No association with radiographic progression	Giles et al. [78, 85]
	Human (serum)	Association with higher insulin resistance Increasing concentrations attenuated the increased risk of coronary calcification related to insulin resistance	Rho et al. [99]
	Human (serum)	Positive correlation with BMI No association with radiographic progression	Klein-Wieringa et al. [79]
	Human (serum, SF)	Serum levels and synovial/serum ratio higher than controls Synovial leptin and synovial/serum leptin ratio in patients with effusion significantly higher than in control subjects with traumatic effusion Serum levels in patients with effusion higher than matched synovial levels Serum levels and synovial/serum ratio significantly correlated with disease activity	Olama et al. [96]

Resistin	Murine (synovial tissue) Human (PBMCs, SF)	Induces an RA-like inflammatory destructive polyarthropathy Marked induction in PBMCs and SF cells of the genes for proinflammatory cytokines Levels in SF higher than matched circulating levels	Bokarewa et al. [69]
	Human (serum)	No association with radiographic progression	Giles et al. [78, 85]
	Human (serum)	Positive correlation with BMI Association with proinflammatory cytokines	Klein-Wieringa et al. [79]

Visfatin	Human (serum)	Positive correlation with IL-6 and TNF Levels significantly higher in ACPA-positive patients Associated with radiographic progression over 4 years	Klein-Wieringa et al. [79]

MMP: matrix metalloproteinase; RASF: rheumatoid arthritis synovial fibroblasts; SF: synovial fluid; OA: osteoarthritis; BMI: body mass index; HMW: high molecular weight; MMW: middle molecular weight; MTX: methotrexate; CRP: C-reactive protein; TNF: tumor necrosis factor; RA: rheumatoid arthritis; PBMCs: peripheral blood mononuclear cells; ACPA: anti-citrullinated protein/peptide antibodies.

**Table 3:** Overview of main experimental and clinical data on adipocytokines in OA.

Adipocytokine	Model	Finding(s)	Authors
Adiponectin	Human (synovial tissue)	Strongly expressed in synovium and adipose tissue Stimulates the release of IL-6 and pro-MMP-1 by synovial fibrobasts	Ehling et al. [62]
	Human (plasma, SF, synovial tissue)	Percentage of HMW adiponectinin SF lower than in plasma, while that of the hexamer form similar and the trimer form higher Up-regulation of tissue TIMP-2 and down-regulation of IL-1*β*-induced MMP-13 in chondrocytes	Chen et al. [113]
	Murine (chondrocytes)	Induces proinflammatory and prodegradative mediators in murine chondrogenic cell lines	Lago et al. [112]
	Human (plasma)	Higher levels than RA patients and healthy controls	Laurberg et al. [84]
	Human (serum)	Higher levels in female patients with erosive than in those with nonerosive disease	Filková et al. [124]
	Human (synovial tissue)	Expression levels of AdipoR1 and AdipoR2 significantly higher in lesional than in nonlesional cartilage Stimulates the release of nitric oxide, MMP-1, -3, and -13 by chondrocytes	Kang et al. [111]
	Human (plasma, SF)	Levels in plasma higher with respect to matched SF Levels in plasma and SF inversely correlated with disease severity	Honsawek and Chayanupatkul [122]
	Human (synovial tissue)	Induces gene expression and synthesis of proinflammatory mediators in fibroblasts to a lesser extent than RASF (adiponectin isoforms)	Frommer et al. [32, 64]
	Human (plasma, cartilage tissue)	Plasma levels and release from cartilage higher in patients with severe disease Plasma levels positively correlated with biomarkers of OA	Koskinen et al. [125]
	Human (serum)	Mean level significantly lower in patients with progression compared with those without progression	Yusuf et al. [123]
	Human (serum)	No difference in levels in patients with radiographic and nonradiographic OA	Choe et al. [126]
	Human (serum)	Higher levels in patients as compared to controls Association of serum levels with female gender and BMI	de Boer et al. [127]

Leptin	Human (serum)	Positive correlation between serum levels and BMI No correlation between serum levels and visual analogue pain scale for the lower-limb afflicted patients and stage of disease	Wisłowska et al. [91]
	Human (SF)	Median concentrations significantly higher in patients compared to controls Positive correlation between SF levels and severity of disease	Ku et al. [120]
	Human (serum)	Association with increased levels of bone formation biomarkers	Berry et al. [119]
	Human (serum)	Mean level slightly higher in patients with progression compared with those without progression	Yusuf et al. [123]
	Human (serum)	Higher levels in patients as compared to controls Association of serum levels with female gender and BMI	de Boer et al. [127]
	Human (cultured chondrocytes)	Stimulates MMP expression in chondrocytes cultured with WAT-conditioned media	Hui et al. [106]
	Human (serum)	Association with prevalent and incident OA	Karvonen-Gutierrez et al. [121]

Resistin	Human (synovial tissue) Mouse (cartilage explants)	Expressed at the same extent of healthy controls Incubation with the conditioned media from monocytes treated with recombinant human resistin caused a dose-dependent proteoglycan release	Lee et al. [116]
	Human (serum)	Mean level not different in patients with progression and those without progression	Yusuf et al. [123]
	Human (serum)	Higher levels in patients with radiographic subchondral erosions than in nonradiographic OA Resistin-treated cartilage released proinflammatory mediators	Choe et al. [126]
	Human (serum)	Higher levels in patients as compared to controls	de Boer et al. [127]

MMP: matrix metalloproteinase; HMW: high molecular weight; SF: synovial fluid; TIMP-2: tissue inhibitor of metalloproteinase-2; RA: rheumatoid arthritis; RASF: rheumatoid arthritis synovial fibroblasts; OA: osteoarthritis; BMI: body mass index; WAT: white adipose tissue.
